# Papillophlebitis Associated With Coexisting Heterozygous Mutations of Factor V Leiden and Methylenetetrahydrofolate Reductase Enzyme-(C677T)

**DOI:** 10.7759/cureus.15081

**Published:** 2021-05-17

**Authors:** Efthalia Ntora, Georgios Dalianis, Chryssa Terzidou

**Affiliations:** 1 Ophthalmology, Private Practice, Athens, GRC; 2 Ophthalmology, Konstantopouleio-Patission General Hospital, Athens, GRC

**Keywords:** papillophlebitis, factor v leiden, methylenetetrahydrofolate reductase mutation, retinal venous disease

## Abstract

We present a rare case of a 22-year-old female patient with floaters in her left eye and atypical orbital pain. Ophthalmic examination revealed optic disc edema with uncomplicated venous congestion (papillophlebitis). Her uncorrected visual acuity was 20/20 in both eyes and visual fields were within normal limits. Biochemical and autoimmune markers were normal, except for Factor V Leiden and methylenetetrahydrofolate reductase enzyme (MTHFR-C677T) heterozygous mutations. Ophthalmoscopic findings resolved completely after one-month treatment with oral methylprednisolone. Genetic analysis has become an integral part of papillophlebitis diagnosis and hematology consultation is essential to prevent future complications.

## Introduction

Papillophlebitis is a rare disease (higher prevalence in women of 20-35 years old), characterized by optic disc edema, retinal venous engorgement, and retinal hemorrhages, mainly located within the peripapillary area, whereas macular edema is rare [1]. The most frequently reported symptoms include mildly affected unilateral visual acuity (VA), without indication of optic nerve conduction defect, resulting in absent relative afferent pupillary defect (RAPD). An enlarged blind spot is common in visual fields (VF). The clinical course is usually benign and the final prognosis is favorable [1,2].

The pathophysiology of papillophlebitis is not clear. However, central vein inflammation at the optic nerve is presumed to be the underlying cause of venous insufficiency [2]. A variety of systemic autoimmune and vascular diseases has also been associated [1], along with risk factors such as pregnancy [3], oral contraceptives (OCs) [4], and thrombophilia [5]. Furthermore, it is advised to screen these patients for hypercoagulable disorders, such as Factor V Leiden (FVL) mutation, hyperhomocysteinemia, and deficiency of vitamin-B6, folic acid, or protein C and S [1].

We describe a case of unilateral papillophlebitis, associated with FVL and methylenetetrahydrofolate reductase enzyme (MTHFR-C677T) heterozygous mutations.

## Case presentation

**Figure 1:**
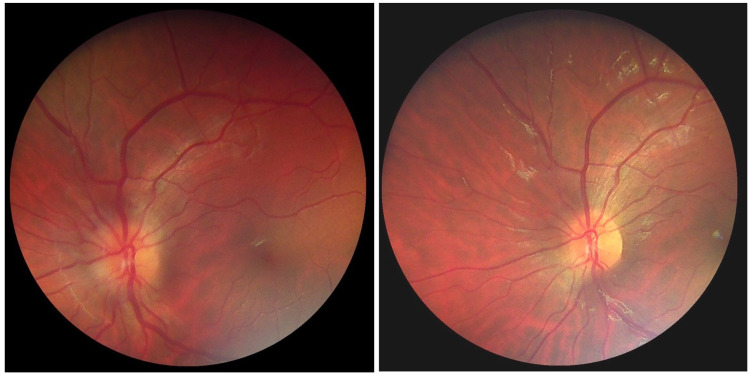
Color fundus photography of the patient at the time of presentation (left) and after one month (right)

A 22-year-old Greek female was evaluated, in an outpatient clinic in March 2020, for left eye (oculus sinister {OS}) floaters and atypical orbital pain for three days. Past medical and family history was not contributory (no use of OCs). Ocular history was unremarkable, without recent ocular trauma. The patient’s uncorrected distance VA was 20/20 in both eyes. The color vision (Ishihara test) was 12/12 in both eyes. Intraocular pressure was 12mmHg in her right eye (oculus dexter {OD}) and 13mmHg in OS. Anterior segment structures appeared normal without pain on ocular movements or RAPD. Fundus examination of OS revealed severe disc edema, retinal venous dilation, and tortuosity. There were no signs of superficial retinal hemorrhages, cotton wool spots, or macular involvement (Figure 1).

**Figure 2:**
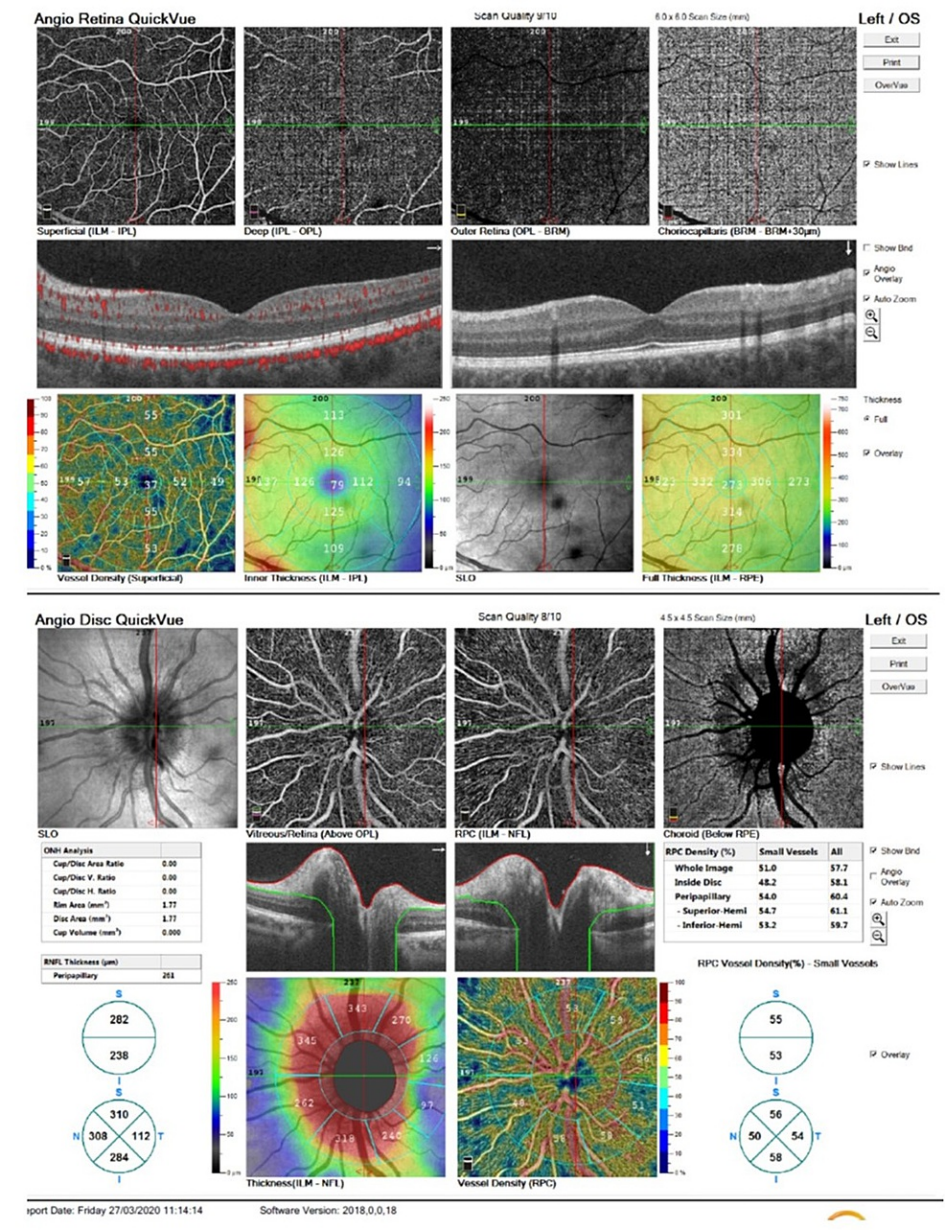
Optical coherence tomography and angiography Prominent optic disc edema, retinal venous engorgement, and congestion on the superficial and deep retinal and choriocapillary plexus scans.

The central macular thickness on optical coherence tomography (OCT) was 271μm in OD and 273μm in OS. Optic nerve head anatomy was evaluated by OCT-angiography (QuickVue-Optovue, USA) since fluorescein-angiography was unavailable at that time. The scan was normal in OD compared to prominent papillary edema and venous congestion on the superficial and deep retinal and choriocapillary plexus scans in OS (Figure 2). Bilateral VF (Humphrey 24-2/SITA-Standard {Zeiss Group, Germany}) was unremarkable and remained normal during follow-up.

The patient’s clinical findings were consistent with papillophlebitis, and she was thoroughly evaluated by biochemical, hematological, hormonal examinations, and autoimmune/inflammatory markers, all of which were within normal limits. A cranial MRI was negative for invasive lesions. Treatment with oral methylprednisolone 50mg/day was initiated, followed by slow tapering. In order to assess thrombophilic risk factors, a molecular genetic test for FVL and MTHFR was performed. A heterozygous mutation of FVL, combined with heterozygous mutation of MTHFR-C677T, was detected (A1298 locus of MTHFR was normal). After one week under methylprednisolone treatment, an improvement in the patient’s symptoms was noticed, along with optic disc edema regression. Interestingly, the orbital pain was rapidly subsided. One month later, venous congestion and disc swelling resolved completely. VA and VF remained normal at six-month follow-up. No further medication was administered in order to prevent future recurrence of papillophlebitis or hypercoagulable complications, except that of the Mediterranean diet (a combination of anti-inflammatory nutrients and foods rich in antioxidants, such as fish, monounsaturated fats from olive oil, fruits, vegetables, whole grains, and legumes/nuts, that have been associated with a beneficial and inverse effect in inflammatory indices, homocysteine, and coagulation process), recommended after hematology consultation [6].

## Discussion

Papillophlebitis is a subtype of central retinal vein occlusion (CRVO) that affects healthy young patients (less than 45 years old), with a favorable prognosis that does not present RAPD [1]. Visual acuity is moderately reduced exhibiting a painless and commonly unilateral pattern [1,2]. Papillary edema is prominent without macular involvement and retinal hemorrhages are located within the peripapillary area counting out all quadrants typically seen in CRVO [7]. Orbital pain is not considered a typical symptom, since there is no known pathophysiological correlation with papillophlebitis. The differential diagnosis of papillophlebitis includes a wide range of vasculopathies [2,3]. In our patient, the findings were consistent with a hypercoagulable state, induced by coexisting heterozygous mutations of FVL and MTHFR-C677T. FVL is the most common hereditary hypercoagulability disorder, and heterozygosity is also considered to be predictive for venous thromboembolism (VTE) as homozygosity [8]. Its clinical expression is influenced by the number of FVL alleles, coexisting genetic and acquired thrombophilic disorders, and circumstantial risk factors [8]. The prevalence of heterozygous FVL mutations in Greece (15%) is among the highest in Europe, with no data available regarding MTHFR polymorphisms [8]. Limited studies in the East Mediterranean region, highlight a relatively frequent co-appearance of heterozygous FVL mutations with MTHFR-C677T in healthy individuals, without, however, an established association between MTHFR-C677T mutations and VTE [9]. The MTHFR-C677T mutation is the most common polymorphism, resulting in hyperhomocysteinemia (in homozygotes), which contributes to a hypercoagulable state and may be a possible cause of papillophlebitis [10]. Nevertheless, our patient had a heterozygous mutation without hyperhomocysteinemia. Rare cases of papillophlebitis with coexisting FVL and prothrombin mutations have been described in the literature, and there are no reports regarding papillophlebitis with combined heterozygosity of FVL and MTHFR-C677T mutations, which makes our case unique [5]. Considering all of these aspects, and based on the molecular genetic analysis, we established a final diagnosis of papillophlebitis, since our findings were consistent with coagulation imbalance. However, it is open to question whether both mutations equally contributed to papillophlebitis pathogenesis or there was a separate pathophysiologic pathway to thrombotic microangiopathy.

Although papillophlebitis, most of the time, resolves spontaneously, oral corticosteroids remain the basic treatment, often combined with the debatable use of anticoagulants [11]. In our case, the patient was sufficiently treated with oral methylprednisolone with excellent tolerability. Orbital pain subsided rapidly without recurrence, despite the fact that it cannot be directly attributed to papillophlebitis. Furthermore, our patient’s papillophlebitis could have been triggered by coronavirus disease 2019/severe acute respiratory syndrome coronavirus 2 (COVID-19/SARS-CoV-2) infection, as it has been described in rare reports [12]. However, the period prevalence of COVID-19 in Greece during March-April 2020 was low, and our patient did not undergo a SARS-CoV-2 test since she had no related symptoms.

## Conclusions

Papillophlebitis is a rarely encountered disease in clinical practice. Individualized management is mandatory, in order to identify and exclude general diseases, including coagulation disorders. Thrombophilia study constitutes a key factor in papillophlebitis investigation. Our case underlines the need for interdisciplinary management in order to prevent oncoming thrombotic events. The presence of FVL combined with MTHFR-C677T heterozygous mutation should be considered as one of the numerous causative factors of papillophlebitis in young healthy patients.
